# Pilomatrix carcinoma of the lacrimal caruncle: a case report

**DOI:** 10.5935/0004-2749.20200029

**Published:** 2020

**Authors:** Ibrahim Inan Harbiyeli, Altan A. Ozcan, Arbil Acıkalin, Emine Ciloglu, Carol L. Shields

**Affiliations:** 1 Department of Ophthalmology, School of Medicine, Cukurova University, Adana, Turkey; 2 Department of Pathology, School of Medicine, Cukurova University, Adana, Turkey; 3 Department of Ophthalmology, University of Health Sciences, Adana City Training and Research Hospital, Adana, Turkey; 4 Ocular Oncology Service, Wills Eye Hospital, Thomas Jefferson University, Philadelphia, PA, USA

**Keywords:** Hair follicle, Hair diseases, Skin neoplasms, Pilomatrixoma, Carcinoma, basal cell, Lacrimal apparatus diseases, Bevacizumab/administration & dosage, Diagnosis, Differential, Humans, Folículo piloso, Doenças do cabelo, Neoplasias cutâneas, Pilomatrixoma, Carcinoma basocelular, Doenças do aparelho lacrimal, Bevacizumab/administração & dosagem, Diagnóstico diferencial, Humanos

## Abstract

A 45-year-old man presented with a 3-month history of a mass located in the caruncle of his right eye. An incisional biopsy had been performed one month prior by another specialist, and the histopathology report showed basal cell carcinoma. The mass was completely excised with a 2 mm safety margin, and the large conjunctival defect was reconstructed with one sheet of amniotic membrane allograft. A histological diagnosis of pilomatrix carcinoma was established. To prevent recurrence after surgery, we added bevacizumab (25 mg/mL, 1.25 mg/mL per drop) eye drops four times per day for three months. At the one-year follow-up, the patient showed no evidence of local recurrence or distant metastasis after initial excision and remains under close follow-up. Pilomatrix carcinoma should be considered in the differential diagnosis of a caruncular mass.

## Abstract (pt)

Um homem de 45 anos apresentou história de massa na carúncula no olho direito durante 3 meses. Uma biópsia incisional foi realizada 1 mês antes por outro especialista e o laudo histopatológico mostrava carcinoma basocelular. A massa foi completamente excisada, com uma margem de segurança de 2 mm, e a grande lesão conjuntival foi reconstruída com uma folha de aloenxerto de membrana amniótica. Foi estabelecido um diagnóstico histológico de carcinoma pilomatricial. Para evitar a recorrência após a cirurgia, adicionamos colírio de bevacizumabe (25 mg/mL, 1,25 mg/mL por gota) quatro vezes ao dia durante três meses. No seguimento de 1 ano, o paciente não apresentou evidência de recidiva local ou metástase distante após a excisão inicial e continua sob acompanhamento próximo. O carcinoma pilomatricial deve ser considerado no diagnóstico diferencial de uma massa caruncular.

## INTRODUCTION

Pilomatricoma is a rare, benign, slow-growing dermal or subcutaneous tumor that occurs most commonly in the head, neck, extremities, and trunk^(1)^. In 1949, Lever and Griesemer^(2)^ proposed that this tumor originates from hair matrix cells. In 1961, Forbis and Helwig^(3)^ pro posed the currently accepted name of “pilomatricoma”. In 1980, Lopansri and Mihm^(4)^ identified malignant trans formations in these tumor cells, and they described these transformations as “pilomatrix carcinoma” (PMC) or “calcifying epitheliocarcinoma of Malherbe.” In the present report, we describe the clinical and histological features in a very rare case of malignant hair follicle tumor involving the conjunctiva. To the best of our knowledge, this is the first report on the PMC of the ocular surface.

## CASE REPORT

**Figure 1:**
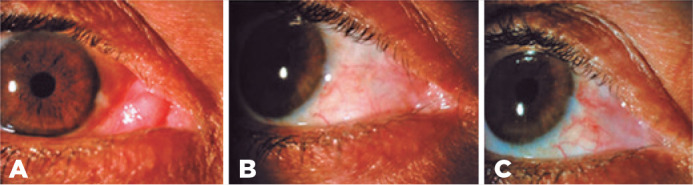
A) Clinical appearance of nonpigmented solid neoplasm involving the medial area of the epibulbar conjunctiva, plica, and caruncle of the right eye. B) Satisfactory cosmetic and functional results at eight weeks after tumor excision and amniotic membrane transplantation. C) No sign of local recurrence at one year after surgery; mild fibrosis is present in the transplanted area.

**Figure 2:**
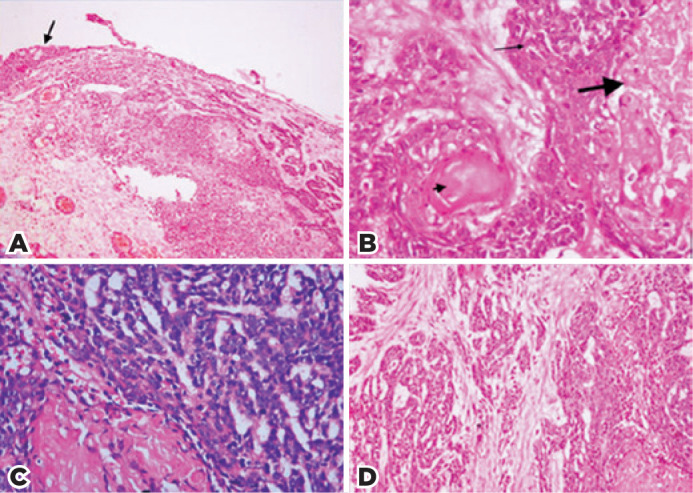
A) Tumor infiltration is present beneath the conjunctiva epithelium (arrow) (H&E, ×40). B) Two types of tumor cells (basaloid [thin arrow] and ghost [thick arrow] cells) can be observed, along with focal keratinization (arrowhead) (H&E, ×400). C) Ghost cells and mitotically active basaloid cells in PMC (H&E, ×400). D) Irregular infiltration of the tumor is present in the desmoplastic stroma (H&E, ×100).

A 45-year-old man presented with a nontender enlarged mass measuring approximately 15 mm × 15 mm that had grown from the lacrimal caruncle of the right eye for more than three months (Figure 1A). He did not complain of pain or discharge from the mass. An incisional biopsy had been performed one month prior by another specialist; the histopathology report showed the presence of basal cell carcinoma. The patient had no notable ophthalmic or medical history. During ocular examination, visual acuity in both eyes was 10/10 (decimal scale), and extraocular movements were normal. The surface of the pinkish mass was smooth without visible vessels, and the well-demarcated lesion was not connected to the surrounding skin. No other lesions were present on the eyelid. The results of the anterior and posterior ocular examinations were normal. Moreover, the patient’s general physical examination results were normal, and there was no evidence of preauricular or submandibular lymphadenopathy. The patient underwent surgical excision and topical chemotherapy. Under general anesthesia, the mass and a 2 mm safety margin were completely excised and sent for histopathological examination. Double freeze-thaw cryotherapy was applied to the conjunctival borders and the base of the mass. The large conjunctival defect was reconstructed with one sheet of amniotic membrane allograft secured with polyglactin sutures. Histopathological examinations revealed the subepithelial irregular infiltration of basaloid cells with hyperchromatic, ovoid, and vesicular nuclei, as well as limited cytoplasm in a trabecular or nested pattern (Figure 2A). Some nests of the basaloid cells showed central keratinization (Figure 2B). In some areas, enucleated “ghost” or “shadow” cells were present with eosinophilic cytoplasm. These cells appeared to have merged with basaloid cell groups (Figures 2B and 2C). Focal giant cell reactions were detected, and no calcification was present. The invasion of the surrounding tissue was observed in desmoplastic stroma (Figure 2D). There were no retraction artifacts between the basaloid cells and stroma. Mitoses were frequently observed (average 20-25 per 10 high-power fields in basaloid areas). No definite vascular or lymphatic permeations were identified. The Ki67 proliferation index was 80%. Immunohistochemical analysis showed diffuse positive reactions for Ber-EP4; however, there were no positive reactions for EMA, p63, S100, CD56, or CK20. A diagnosis of PMC was made on the basis of patient age, tumor localization, and histomorphological findings. The surgical margins were free of tumor cells. The results of complete blood cell count, renal and liver function tests, chest x-ray, and neck and abdomen ultrasound investigations were normal. The patient provided informed consent for further treatment; thus, we administered bevacizumab (25 mg/mL, 1.25 mg/mL per drop) eye drops four times per day for three months to prevent recurrence after surgery. At the one-year follow-up after initial excision, the patient showed no evidence of local recurrence or distant metastasis. The patient continues to be closely monitored (Figures 1B and 1C).

## DISCUSSION

The most common sites for PMCs are the head and neck, followed by the upper extremities, trunk, and lower extremities^(5)^. In a review by Sia et al.^(6)^, 6 cases were reported to be PMCs among the 16 cases involving hair follicle malignancies in the periorbital region. In that review, the upper lid was the most commonly affected site by malignant hair follicle tumors, followed by the lower lid, eyebrow, and medial canthal region. The lacrimal caruncle has a nonkeratinized epithelial lining that is similar to the conjunctival epithelium^(7)^. Developmentally, it constitutes a part of the lower lid; therefore, it contains hair follicles, sebaceous glands, and sweat glands. It also harbors accessory lacrimal tissue. Hence, neoplasms that may arise from the skin, conjunctiva, and lacrimal gland may develop in the lacrimal caruncle^(7)^. None of the cases identified in the PubMed database reported on the primary hair follicle tumor of the lacrimal caruncle.

Conjunctival involvement is not a typical finding in hair follicle malignancies. Lee et al.^(8)^ reported a case of trichilemmal carcinoma of the upper eyelid in a 51-year-old man. They reported that the mass completely penetrated the inner side of the upper lid and was present on the conjunctival side during slit-lamp examination. To the best of our knowledge, the present report is the first description of the PMC of the ocular surface involving the conjunctiva. In our case, PMC was originally misdiagnosed as basal cell carcinoma after incisional biopsy; it was later diagnosed as PMC on the basis of the excised specimen. This misdiagnosis was caused by the lack of clear histologic criteria and the lack of a specific marker to distinguish this neoplasm from other matrical tumors ^(5)^.

Histopathologically, PMCs can be distinguished from benign pilomatricoma, trichoblastic carcinoma, and basal cell carcinoma owing to matrical differentiation. Basal cell carcinoma presents with infiltrating islands of palisading basaloid cells with shadow cells. Histological examination can be challenging, and malignant pilomatricoma with many basophilic basal cells is often mistaken for basal cell carcinoma^(9)^. Differentiation is dependent upon the observation of retraction spaces between neoplastic cells and stroma^(5,9)^. In the present case, there were no retraction artifacts between basaloid cells and stroma. Furthermore, proliferating pilomatricoma, which is a pathological variant of pilomatricoma that consists predominantly of mitotically active basaloid cells, should be included in the differential diagnosis. In such cases, poor circumscription, an asymmetrical appearance, atypical mitoses, and lymphovascular invasion are features that favor a diagnosis of PMC^(10)^.

PMC can exhibit local aggressive behavior with a tendency toward recurrence. Thus, we administered topical chemotherapy to prevent recurrence even though the surgical margins were free of tumor cells. To the best of our knowledge, this is the first reported case of PMC of the ocular surface; therefore, there is no standard topical chemotherapy protocol for similar cases. It has recently been reported that topical bevacizumab may reduce tumor size prior to surgery or may completely cure the tumor in ocular surface squamous neoplasia^(11,12)^. Additionally, the systemic application of bevacizumab is a treatment option in PMC when local recurrence and distant metastasis are detected^(13)^. Although the use of topical bevacizumab as an adjuvant therapy after the surgical treatment of ocular surface neoplasms is not a proven treatment option, it is regarded a good and safe option on the basis of the literature described above. We have not observed any side effects after three months of continuous treatment with topical bevacizumab.

Local recurrences and metastatic disease have been documented after the simple excision of these types of lesions^(6)^. Therefore, the creation of a sufficiently wide excision with a tumor-free margin is important during histopathological examination. In our case, after considering the clinical appearance of surgical margins and the results of the previous biopsy, we concluded that the resection of the tumor with a 2 mm margin of safety would ensure its complete removal. A wider excision could have been made after pathological examination, but there was a high risk of inadvertently damaging the canalicular system because of the proximity of the primary lesion to this system. Furthermore, the patient would have been required to undergo general anesthesia because he could not tolerate surgery under local anesthesia, and it would have been more difficult to make precise excisions because the tumor margin would be obscured following two previous surgeries in the same area. The patient was informed of the current situation and decided not to undergo a second surgical procedure. Consequently, a second surgery was not performed. Topical chemotherapy was initiated, and the patient underwent continuous monitoring.

This case highlights the rare potential for conjunctival lesions to have unusual origins with potentially serious consequences. To make accurate excisions during the initial surgery, frozen section examinations should be performed during surgery, and differential diagnosis should be made with the inclusion of conditions such as PMC.
